# The Mechanism of Calcium-Induced Inhibition of Muscle Fructose 1,6-bisphosphatase and Destabilization of Glyconeogenic Complex

**DOI:** 10.1371/journal.pone.0076669

**Published:** 2013-10-11

**Authors:** Dariusz Rakus, Agnieszka Gizak, Andrzej A. Kasprzak, Marek Zarzycki, Ewa Maciaszczyk-Dziubinska, Andrzej Dzugaj

**Affiliations:** 1 Department of Animal Molecular Physiology, Wroclaw University, Wroclaw, Poland; 2 Department of Biochemistry, Nencki Institute of Experimental Biology, Warsaw, Poland; 3 Depatment of Genetics, Wroclaw University, Wroclaw, Poland; 4 Department of Genetics and Cell Physiology, Wroclaw University, Wroclaw, Poland; Institute of Enzymology of the Hungarian Academy of Science, Hungary

## Abstract

The mechanism by which calcium inhibits the activity of muscle fructose 1,6-bisphosphatase (FBPase) and destabilizes its interaction with aldolase, regulating glycogen synthesis from non-carbohydrates in skeletal muscle is poorly understood. In the current paper, we demonstrate evidence that Ca^2+^ affects conformation of the catalytic loop 52–72 of muscle FBPase and inhibits its activity by competing with activatory divalent cations, e.g. Mg^2+^ and Zn^2+^. We also propose the molecular mechanism of Ca^2+^-induced destabilization of the aldolase–FBPase interaction, showing that aldolase associates with FBPase in its active form, i.e. with loop 52–72 in the engaged conformation, while Ca^2+^ stabilizes the disengaged-like form of the loop.

## Introduction

Over the last years a large body of evidence has shown that glycolytic enzymes in a variety of cells may form metabolically active macromolecular complexes [1, for a review see: 2,3], whose stability is regulated directly and indirectly by calcium ions [4], [5] and glycolytic intermediates [6], [7]. Such association not only alters the regulatory properties and the kinetics of glycolytic enzymes [8], but may also facilitate the channeling of substrates between metabolically sequential enzymes increasing the velocity of the glycolytic pathway [9, for a review see: 2,3].

For years it was a common belief that lactate produced in glycolysis in a contracting muscle is transported via the blood stream to the liver where it is converted to glucose, which is subsequently transported back to the muscle (“the Cori cycle”). However, evidence has accumulated that in skeletal muscle up to 50% of lactate is converted to glycogen [10]. This suggests that glyconeogenesis, glycogen synthesis from non-carbohydrates, significantly contributes to the maintenance of energy stores in vertebrate striated muscle. Additionally, it has been demonstrated that the glyconeogenic enzymes also form protein complexes that may enable substrate channeling [11].

Fructose 1,6-bisphosphatase (FBPase; **EC 3.1.3.11**) is a key enzyme of gluco- and glyconeogenesis. It catalyzes the hydrolysis of fructose 1,6-bisphosphate (F1,6P_2_) to fructose 6-phosphate (F6P) and inorganic phosphate, in the presence of divalent metal ions such as Mg^2+^, Mn^2+^, Co^2+^ or Zn^2+^ [12], [13]. The enzyme is activated by several monovalent cations (e.g. K^+^, NH_4_^+^, Tl^+^) [14], inhibited competitively by fructose 2,6-bisphosphate (F2,6P_2_) and allosterically by adenosine 5′-monophosphate (AMP) and nicotinamide adenine dinucleotide (NAD) [12], [15]. FBPase is also inhibited – in an unknown manner – by Ca^2+^ [16].

Vertebrate genomes contain two distinct genes – FBP1 and FBP2, coding two FBPase isozymes. A protein product of the FBP1 gene – liver FBPase, is expressed mainly in gluconeogenic organs, where it functions as a regulator of glucose synthesis from non-carbohydrates. The muscle FBPase isozyme is the sole FBPase isozyme in striated muscle and it is widely expressed in non-gluconeogenic cells [17].

Mammalian muscle FBPase in comparison to the liver isozyme, is about 100 times more susceptible to the action of the allosteric inhibitors – AMP and NAD, and about 1,000 times more sensitive to inhibition by Ca^2+^ [11], [13], [15], [16] – the most potent activator of glycolysis in striated muscle. Moreover, calcium not only inhibits muscle FBPase but also disrupts the Z-line based FBPase–aldolase complex in striated muscles, blocking the re-synthesis of glycogen during high-intensity exercise [18], [19]. However, a mechanism of this action by Ca^2+^ is unclear. Mammalian FBPase is a homotetramer [20] and exists in at least two conformations: R (catalytically active) and T (inactive), depending on the relative concentrations of the enzyme effectors [20], [21]. A proposed mechanism governing the regulation and catalysis of FBPase involves three conformational states of loop 52–72 called engaged, disengaged, and disordered [22]. The enzyme is active (R) if loop 52–72 can switch between its engaged and disordered conformations [22]–[24]. Divalent cations such as Mg^2+^, Mn^2+^, or Zn^2+^ together with F6P or F1,6P_2_ stabilize the engaged state of the loop and the R-state of the tetramer. Binding of AMP to FBPase induces the conversion of the enzyme into the T-state which is hypothesized to stabilize the disengaged, inactive conformation of loop 52–72 [22], [24].

The results of our previous studies suggested that residues involved in the activation of FBPase by Mg^2+^ are also involved in the inhibition of the enzyme by Ca^2+^ [25]. Nonetheless, a mode in which the binding of Ca^2+^ affects the conformation of loop 52–72 remained unclear.

Thus, the primary aim of our present work was to investigate the molecular mechanism of the inhibition of muscle FBPase by Ca^2+^. Here, we demonstrate the effect of Ca^2+^ on the conformation of loop 52–72 and provide evidence that Ca^2+^ inhibits muscle FBPase competitively to Mg^2+^.

We also show that in striated muscle, aldolase associates with FBPase in its active form, i.e. with loop 52–72 in the engaged conformation, while Ca^2+^ stabilizes the disengaged-like form of the loop and disrupts the FBPase-aldolase association. To the best of our knowledge, this is the first paper describing the mechanism of muscle FBPase inhibition and FBPase-aldolase complex regulation by calcium ions and providing an explanation of calcium-dependent regulation of glyconeogenic complex activity in striated muscles.

## Materials and Methods

This study was carried out in strict accordance with the recommendations of the Polish Committee on the Ethics of Animal Experiments. The protocol was approved by the II Local Scientific Research Ethical Committee, Wroclaw University of Environmental and Life Sciences (Permit Number 118/2010).

### Mutagenesis, Protein Expression and Purification

The Escherichia coli strain XL1-Blue MRF’Kan (Stratagene, La Jolla, USA) was used for transformation, propagation and isolation of plasmids as well as for expression of recombinant FBPase, and was grown at 37°C in Luria Broth, supplemented with 100 µg/mL ampicillin [26].

Plasmid isolation, DNA restriction endonuclease analysis, ligation and transformation were performed as described [26]. Either a Qiaprep spin miniprep kit or a Qiaquick gel extraction kit (Qiagen, Germany), was used to prepare plasmid DNA for restriction enzyme digestion, sequencing, and recovering DNA fragments from agarose gels. The sequence of the mutant gene product was confirmed by Sanger DNA sequencing on an ABI 377 sequencer using the Big Dye Terminator Cycle Sequencing Kit (Applied Biosystems, USA).

Mutation in the sequence of human muscle FBPases was introduced by site-directed mutagenesis using the QuikChange® Lightning Site-Directed Mutagenesis Kit (Agilent Technologies). Primers used to introduce the Tyr^57^Trp mutation into the muscle FBPase were:

Tyr^57^TrpFor 5′-GTCTGGCCCACCTGTGGGG AATCGCAGGAAG-3′ and

Tyr^57^TrpRev 5′-CTTCCTGCGATTCCCCACAGGTGGGCCAGAC-3′.

Protein expression and purification were performed as described previously [15]. Protein purity and concentration throughout the purification procedure were monitored by SDS-PAGE and Bradford assay, respectively.

### Steady-state Fluorescence and Enzyme Kinetic Measurements

Fluorescence data were collected using a Fluorolog 3 (Spex-Horiba) fluorometer. To avoid exciting tyrosyl side chains, an excitation wavelength of 290 nm was used. Emission spectra were recorded from 300 to 420 nm, using a spectral slit width of 2 nm for the excitation and 3 nm for the emission monochromator. To minimize Trp photobleaching, the spectra were acquired using a fast scanning mode (2.5 nm per step, 0.5 s integration time). Before measurements, all samples were carefully temperature-equilibrated. Enzyme concentration was 0.1 mg/mL (2.7 nmol of monomers/mL) in 50 mM MOPS buffer, pH 7.0, 37°C. Conditions under which specific spectra were recorded are provided in the text, tables, and figure legends. Control experiments demonstrated that, if several spectra of FBPase were taken without any additions, they were completely superimposed.

All kinetic experiments were performed at pH 7.0 and 37°C using a glucose-6-phosphate isomerase – glucose-6-phosphate dehydrogenase coupled spectrophotometric assay [27] and 50 mM MOPS buffer, pH 7.0, 37°C.

The forward FBPase reaction was started with the saturating concentration of F1,6P_2_ (50 µM). One unit of enzyme activity is defined as the amount of the enzyme that catalyzes the formation of 1 µmol of product per minute.

The reverse FBPase reaction was measured in a mixture containing: 50 mM MOPS, 150 mM KCl, 2.25 mM MgCl_2_, 0.25 mM EDTA, 5 mM fructose-6-phosphate, 5 mM KPi; 0.1 mM NADH, 5 U/mL of rabbit muscle aldolase, 10 U/mL of triose-3-phosphate isomerase and 10 U/mL of glycerol-3-phosphate dehydrogenase, pH 7.0, 37°C.

Spectrophotometric measurements were performed with the Agilent 8453 diode array spectrophotometer. Determination of kinetic parameters such as the dissociation constant of the enzyme-substrate complex (*K_s_*), the inhibition constant of FBPase by its substrate (*K_is_*), β and the catalytic rate constant (*k_cat_*) were performed assuming the model of partial non-competitive inhibition by substrate, which assumes that F1,6P_2_ may associate with the canonical active site and the inhibitory site, which also catalyses the hydrolysis of the substrate but the *k_cat_* is lower [27]. The overall velocity at which product is formed may be written as followed:
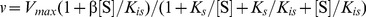
(1)


Where: *K_s_* is an enzyme-substrate dissociation constant, *K_is_* is the inhibition constant of FBPase by substrate and β is the ratio of *k_cat_* when substrate binds to the inhibitory site to *k_cat_* when substrate binds only to the active site.

The values of *K_i_* and *n* for AMP and *K_a_* and *n* for Mg^2+^ were calculated using the Hill equation [28].

**Figure 1:**
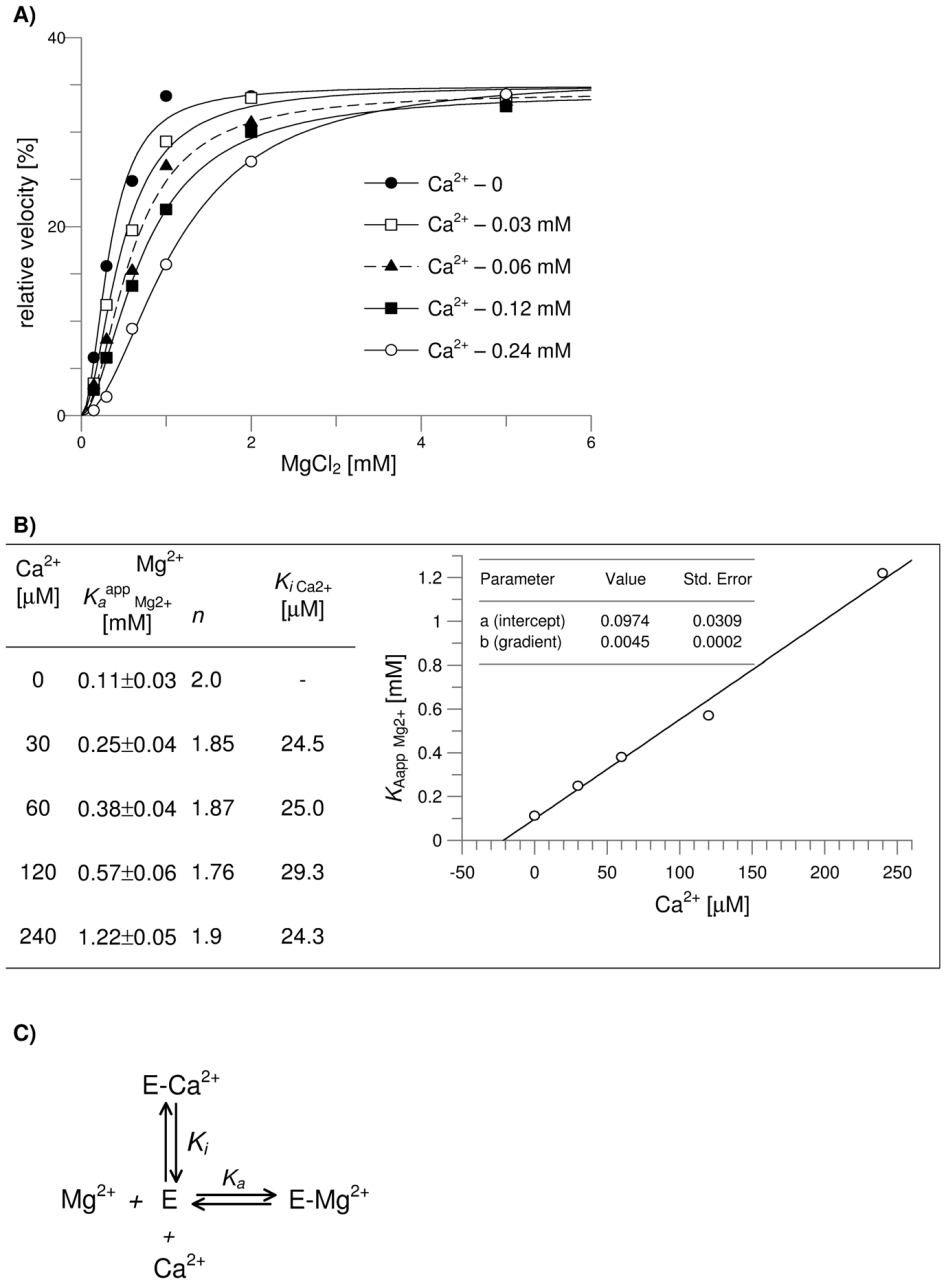
The effect of Ca^2+^ on kinetic parameters of wild-type and mutated form of muscle FBPase. **A)** Activation of the Tyr^57^Trp muscle FBPase mutant by Mg^2+^ in the presence of various concentrations of calcium. **B)** Calcium-induced increase in apparent dissociation constant for Mg^2+^ (*K_a_* ^app^_Mg2+_) does not affect the value of dissociation constant for Ca^2+^ (*K_i_*_ Ca2+_). Hill constant (*n*) is given for the activation by Mg^2+^. The plot shows that the increase in *K_a_* ^app^_Mg2+_ is a linear function of Ca^2+^ concentration. The average value of *K_i_* for Ca^2+^ calculated from the plot (*K_i_*_ Ca2+_) equals to 21.65 µM. **C)** The mechanism that produce competition between magnesium and calcium ions. From this, the equation describing the competitive inhibition is: 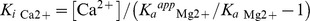, where *K_a_* ^app^_Mg2+_ is the apparent activator’s (Mg^2+^) dissociation constant and *K_a_*_ Mg2+_ is the dissociation constant for Mg^2+^ as determined in the absence of Ca^2+^.

The effect of Ca^2+^ on the activation of FBPase by Mg^2+^ was analyzed using the Michaelis–Menten kinetics-derived equation describing competitive inhibition (Fig. 1 C) [28].

In brief, the effect of competitive inhibition by Ca^2+^, in respect to Mg^2+^, may be written as (2):
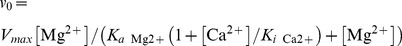
(2)where: *v_0_* is reaction velocity, *V_max_* is the maximal velocity, [Ca^2+^] is the concentration of the inhibitor (Ca^2+^), [Mg^2+^] is Mg^2+^ concentration, and *K_a_*_ Mg2+_ is the dissociation constant for Mg^2+^ determined in the absence of the inhibitor.

From this (3):
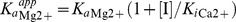
(3)where *K_a_* ^app^_Mg2+_ is apparent activator’s (Mg^2+^) dissociation constant, and *K_i_*_ Ca2+_ is an inhibitor’s (in this case, Ca^2+^) dissociation constant.

Equation (2) may be rearranged as follows (4):
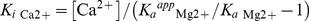
(4)


### Fluorescent Labeling

Fluorescently labeled wild-type (WT) muscle FBPase and the Tyr^57^Trp mutant of muscle FBPase were obtained by modification with tetramethyl-rhodamine isothiocyanate (TRITC, isomer B) and fluorescein isothiocyanate (FITC), respectively, as described by Goding [29]. The lack of proteolysis of fluorescently labeled protein was checked by 10% SDS-PAGE. The number of fluorochrome molecules conjugated to the enzyme was estimated spectrophotometrically. FBPase monomer bound in an average 1.5 molecules of TRITC or FITC.

### The Protein Exchange

23-day old female Wistar rat was obtained from the Department of Pathological Anatomy, Wroclaw Medical University. The animal was euthanized by decapitation, in accordance with the rules of The Scientific Research Ethical Committee. The gastrocnemius muscle was immediately dissected and single muscle fibers were isolated, as described by Kraft et al. [30]. The protein exchange method, described by Gizak et al. [16], was used to localize the TRITC-labeled WT FBPase and the FITC-labeled Tyr^57^Trp mutant in the presence of various concentrations of Ca^2+^.

Before the experiment, the labeled proteins were dialyzed for 5 h against a relaxing solution (10 mM imidazole, 2 mM MgCl_2_, 1 mM EGTA, 1 mM ATP, 20 mM creatine phosphate, 2 mM dithiothreitol, and 106 mM potassium propionate; pH 7.0, at 4°C). The fibers were incubated overnight at 4°C in a drop (100 µL) of the relaxing solution with 0.04 mg/mL of WT or Tyr^57^Trp FBPase. All fibers were washed several times with the relaxing solution. Directly before microscopy (Olympus FluoView 1000 confocal microscope), the fibers were immersed in the relaxing solution supplemented with 0, 10, or 200 µM Ca^2+^ and mounted on slides.

To avoid cross-talk between the channels, the Sequential Scan option was used to observe double-stained fibers.

## Results

### The Kinetics of the Wild-type and Tyr^57^Trp Mutant of Human Muscle FBPase

The wild-type and mutant proteins were purified to homogeneity, as determined with the Coomassie-stained SDS-PAGE (data not shown). As mammalian FBPases have no tryptophan, introduction of this residue with site-directed mutagenesis provides a convenient tool for a spectroscopic study of the enzyme’s conformational response to its effectors.

The mutation of tyrosine to tryptophan (Tyr^57^Trp) did not affect significantly the kinetic properties of FBPase, except for the *K_i_* values (inhibitor’s dissociation constant) for inhibition by Ca^2+^ and AMP (Table 1). A similar phenomenon (reduced inhibition of Tyr^57^Trp mutant of liver FBPase by AMP) was observed by Nelson et al. [24], who hypothesized that it resulted from the lowered ability of loop 52–72 to adopt a disengaged conformation, correlated with an inactive form of the enzyme.

**Table 1:** The kinetic properties of the wild-type and Tyr^57^Trp mutant form of human muscle FBPase.

	Mg^2+^		Ca^2+^		AMP		F1,6P_2_			
	*K_a_* [µM]	*n*	*K_i_* [µM]	*n*	*K_i_* [µM]	*n*	*K_s_* [µM]	*K_is_* [µM]	β	*k_cat_* (s^−1^)
WT muscle	116±4	1.8±0.3	0.98±0.19	1.49±0.12	0.031±0.001	1.9±0.1	3.6±0.5	35±7	0.63±0.08	21.7±2.2
Tyr^57^Trp	129±3	2.0±0.4	21.0±0.1	1.84–0.20	0.81±0.05	2.0±0.2	4.1±0.4	29±8	0.57±0.10	24.7±2.9

The dissociation constant of the enzyme-substrate complex (*K_s_*), the inhibition constant of FBPase by its substrate (*K_is_*) and β values were calculated assuming the model of partial noncompetitive inhibition by substrate [18].

The Hill equation was used to calculate dissociation constants for Mg^2+^, Ca^2+^ and AMP.

*K_i_* is a dissociation (inhibitory) constant for AMP or Ca^2+^, *K_a_* is a dissociation (activatory) constant for Mg^2+^ and *n* is the Hill constant.

The mean values and respective standard error calculated from 3 independent experiments are presented in the Table.

Although the *K_i_* value for AMP increased about 5–6 times relatively to the wild-type muscle FBPase, there was no significant change in the cooperative mechanism of the inhibition by AMP – the Hill constant was about 2, for both the wild-type muscle FBP and for Tyr^57^Trp mutant.

On the other hand, a significant desensitization of the mutant to Ca^2+^ action was correlated with a slight increase in cooperativity as compared to the wild-type muscle FBPase (Table 1). Although the mechanism leading to this small change in the cooperativity is unclear, the relatively weak Ca^2+^ sensitivity of the mutant presented an opportunity to examine the competition between Mg^2+^ and Ca^2+^. As shown in Fig. 1, an increase in Ca^2+^ concentration resulted in decreased activation of the Tyr^57^Trp mutant by Mg^2+^ (Fig. 1A). The changes in the apparent *K_a_* for Mg^2+^ (*K_a_* ^app^_ Mg2+_) had practically no impact on the maximal velocity of the reaction (Fig. 1A) and the cooperativity of the activation (Fig. 1B – data in the table ).

Thus, the observed increase in *K_a_* ^app^_ Mg2+_ strongly suggests that the effect of Mg^2+^ and Ca^2+^ was competitive. In fact, we found that Ca^2+^-dependent changes in *K_a_* ^app^_Mg2+_ were described well by the competitive binding model between the cations (Fig. 1B). The increase in *K_a_* ^app^_ Mg2+_ was a linear function of Ca^2+^ concentration, which confirms that the activation by Mg^2+^ is competitive to Ca^2+^ inhibition. The average value of *K_i_*_ Ca2+_, calculated from the plot of *K_a_* ^app^_Mg2+_ versus Ca^2+^ concentration, was indistinguishable from that presented in Table 1 (determined using the Hill equation and the data on the effect of increasing Ca^2+^ on reaction velocity).

### Fluorescent Studies

As shown by Nelson et al. [24], site-directed mutation introducing tryptophan into loop 52–72 (mutation Tyr^57^Trp) is allows to study the influence of FBPase effectors on the conformation of the loop.

**Figure 2:**
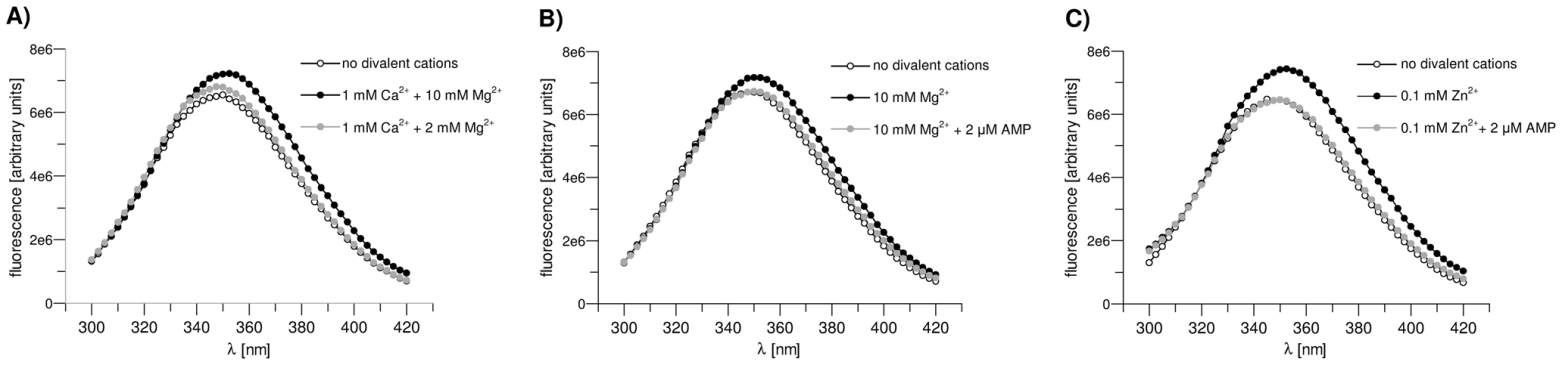
Fluorescence spectra of the Tyr^57^Trp mutant under different ligation conditions. **A)** Enzyme under R-state conditions of ligation (5 mM F6P and 5 mM KPi) in the presence of various concentration of Ca^2+^ and Mg^2+^. **B)** Enzyme under R-state conditions of ligation (5 mM F6P and 5 mM KPi) in the presence of various concentration of Mg^2+^ and under T-state conditions of ligation (5 mM F6P, 5 mM KPi, and 2 µM AMP) in the presence of Mg^2+^. **C)** Enzyme under R-state conditions of ligation (5 mM F6P and 5 mM KPi) in the presence of various concentration of Zn^2+^ and under T-state conditions of ligation (5 mM F6P, 5 mM KPi, and 2 µM AMP) in the presence of Zn^2+^. The final emission spectra do not depend on the sequence of the ligands addition.

All fluorescent spectra in Fig. 2 were acquired in the presence of the substrates of the synthetic reaction of FBPase: 5 mM F6P and 5 mM KPi. It was previously demonstrated that liver FBPase may synthesize F1,6P_2_ from F6P and Pi and that the velocity of this reverse reaction is about 1% of the forward one [31]. In this study, the velocity of the synthetic reaction catalyzed by the muscle FBPase Tyr^57^Trp mutant was very low (∼0.01 U/mg protein) compared to the hydrolytic reaction (∼40 U/mg protein). Nonetheless, the synthetic activity of the mutant was regulated by AMP and divalent metal cations in a similar manner to its hydrolytic activity (Table 1 and 2) making the mutant a convenient model to study structural changes of muscle FBPase.

**Table 2:** The influence of FBPase effectors on the reverse reaction of FBPase Tyr^57^Trp mutant.

effector	Relative velocity [%]
2 mM Mg^2+^	100±3
1 µM AMP	85±7
2 µM AMP	50±8
5 µM AMP	15±6
0.1 mM Ca^2+^ (Mg^2+^ = 2 mM)	77±4
0.5 mM Ca^2+^ (Mg^2+^ = 2 mM)	32±5
2 mM Ca^2+^ (Mg^2+^ = 2 mM)	9±2
25 µM Zn^2+^ (Mg^2+^ = 0)	84±7
100 µM Zn^2+^ (Mg^2+^ = 0)	79±8

The mean values and respective standard error are presented in the Table. The measurements were repeated in triplicate.

In the absence of FBPase substrates, the addition of activatory metal cations did not result in an observable increase in Trp^57^ fluorescence (data not shown). Likewise, there was no change in the fluorescent emission spectrum when FBPase substrates (F6P and KPi) were added to the enzyme in the absence of the activatory metal cations (data not shown). Both complexes, FBPase-activatory metal cations and FBPase-substrates, are inactive because loop 52–72 cannot adopt the engaged conformation, although the tetramer is in R-state.

The addition of activatory metal cations to F6P- and KPi-saturated FBPase caused an increase in the fluorescence intensity of Trp^57^ by about 11–15% and a red shift of λmax, from 348 nm to about 351 and 353 nm for Mg^2+^ and Zn^2+^, respectively (Fig. 2, Table 3). Evidently, these changes are correlated with the activation of the enzyme by divalent cations (Fig. 2, Table 2 & 3) and hence, with a conformational shift of loop 52–72 from its disengaged towards the engaged state.

**Table 3:** Fluorescence emission from ligated complexes of Tyr^57^Trp FBPase.

			2 µM AMP		1 mM Ca^2+^		2 mM Ca^2+^	
cation	*F*	λmax (nm)	*F*	λmax (nm)	*F*	λmax (nm)	*F*	λmax (nm)
none	**100%**	348	99.5%	348	100%	348	100%	348
Mg^2+^ – 2 mM	104%•	348	100%**	348	104%	349	101%**	348
Mg^2+^ – 10 mM	107.5%•	350○	101%**	349	109%*	350	102.5%**	349
Mg^2+^ – 20 mM	111.4%•	351○	nd	nd	112.7%	353	nd	nd
Zn^2+^ – 25 µM	108.7%•	350○	nd	nd	nd	nd	nd	nd
Zn^2+^ – 50 µM	112.6%•	353○	nd	nd	nd	nd	nd	nd
Zn^2+^ – 100 µM	115.4%•	353○	102.3%**	349*	114.9%	353	103.1%**	351*

*F* – relative mean fluorescence emission at maximum from the Tyr^57^Trp mutant in the presence of F6P (5 mM) and KPi (5 mM).

λmax – a mean λmax from three independent experiments. Mean values from three independent experiments are presented in the table.

An increasing concentration of Mg^2+^ and Zn^2+^ (down to the first column) induces significant (p<0.005) changes in the fluorescence (full circle) and a slight red-shift (empty circle – p<0.05) as compared to the fluorescence measured in the absence of the cations.

Asterisk indicates a significant difference (** - p<0.005, * - p<0.05) in fluorescence upon addition of AMP or Ca^2+^ to the enzyme saturated with various concentrations of Mg^2+^ and Zn^2+^.

nd - not detected.

Addition of AMP or Ca^2+^ at concentrations completely inhibiting muscle FBPase to any of the FBPase-F6P-Pi-activatory cations complexes resulted in a decrease in fluorescence intensity and in a blue shift of λmax (Fig. 2 B–C, Table 3) reversing the changes induced by Mg^2+^ or Zn^2+^. In fact, the emission spectra of these FBPase complexes were nearly identical to those recorded in the absence of the activatory metal cations (Table 3). This indicates that the inactive, saturated with AMP or Ca^2+^, or depleted of the activatory cations FBPase adopts a disengaged-like conformation of loop 52–72.

### The Effect of Calcium on the Subcellular Localization of Various Forms of Muscle FBPase

**Figure 3:**
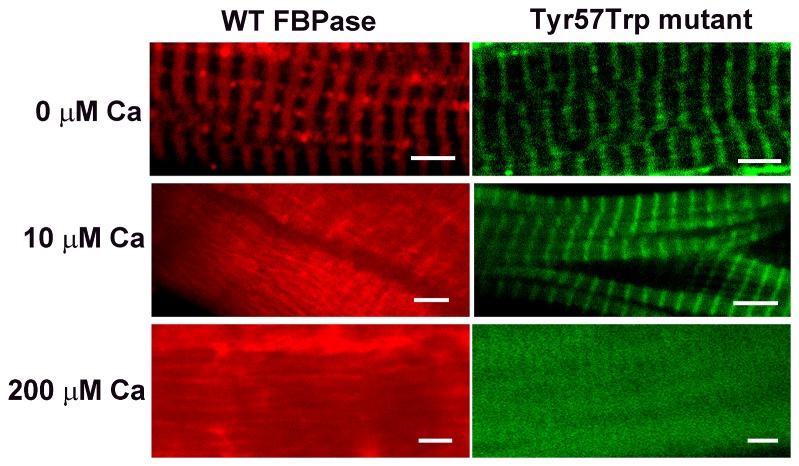
Effect of calcium on the association of the wild-type and Tyr^57^Trp mutant of human muscle FBPase with sarcomeric Z-line. In control conditions, TRITC-labeled WT FBPase (red) and FITC-labeled Tyr^57^Trp mutant (green) accumulates on the sarcomeric Z-lines. In the presence of 10 µM Ca^2+^, WT FBPase dissociated from the Z-line but the Tyr^57^Trp mutant remained bound to the sarcomeric structures. 200 µM Ca^2+^ disrupted interactions of both the proteins with Z-line.

Since it is known that Ca^2+^ concentrations that inhibit muscle FBPase also influence its interaction with its cellular binding partners [16], [32], we tested the influence of elevated [Ca^2+^] on the localization of WT FBPase and the Tyr^57^Trp mutant in skeletal muscle fibers. TRITC-labeled WT muscle FBPase accumulated on the sarcomeric Z-lines (Fig. 3; [25]), as did the FITC-labeled Tyr^57^Trp muscle FBPase mutant. In the presence of 10 µM Ca^2+^, WT FBPase dissociated from the Z-line. In the same conditions, the Tyr^57^Trp mutant remained bound to the sarcomeric structures. Preincubation of muscle fibers with 200 µM Ca^2+^ resulted in the disruption of the Tyr^57^Trp mutant–Z-line interactions and diffused the localization of the protein (Fig. 3). During the whole experiment, interactions of muscle aldolase (a binding partner of muscle FBPase) with the sarcomeric structures remained undisrupted (File S1; Fig. S1).

## Discussion

Muscle glyconeogenesis proceeds only if muscle FBPase and muscle aldolase form a complex in the region of the sarcomeric Z-line [19]. Stability of this complex is down-regulated by cytosolic concentration of Ca^2+^ [32]. Thus, glycolysis and glyconeogenesis are inversely regulated by changes in the concentration of this cation [2], [19], [33].

The mode in which Ca^2+^ destabilizes the glyconeogenic complex and inhibits free muscle FBPase is unknown. In the present paper, we used the muscle FBPase Tyr^57^Trp mutant to clarify this mechanism.

The role of divalent ions, like Mg^2+^, Mn^2+^ and Zn^2+^, in hydrolysis of F1,6P_2_ by liver FBPase has been investigated by Fromm’s group [22]–[24]. They found that these metals stabilize the catalytic loop 52–72 of the enzyme in the engaged conformation, which equates with the catalytically active state of FBPase. In contrast to these cations, Ca^2+^ inhibits FBPase [16], [25]. While the inhibition of the liver isozyme does not seem to have any physiological function (*K_i_*>1 mM), the inhibition of muscle FBPase is much stronger (*K_i_*≈1 µM), and it plays an important role in regulating the isozyme activity *in vivo* [16], [25]. Recently, it has been reported that residue 69 (glutamine or glutamic acid in the liver and muscle FBPase, respectively) as well as the differing amino-acid compositions of the N-terminal region of these isozymes are responsible for the different sensitivities of the two FBPases to Ca^2+^ inhibition [25], [34]. It has also been shown that mutation of glutamic acid 69 to alanine decreases the sensitivity of muscle FBPase to inhibition by Ca^2+^ and to activation by Mg^2+^ [34]. However, it has remained unclear whether Ca^2+^ competes with Mg^2+^ for the binding to FBPase and inhibits FBPase activity thus preventing the release of the enzyme product or whether Ca^2+^ stabilizes the catalytic loop 52–72 in a new conformation that does not support catalysis. The results of our kinetic studies demonstrate that Ca^2+^ competes with Mg^2+^ for the binding to muscle FBPase. Ca^2+^ not only displaces Mg^2+^ from the active site but also affects the active, engaged conformation of loop 52–72. Fluorescence studies with Trp57 reporter probe have shown that the association of Ca^2+^ with FBPase correlates with the inactive, disengaged-like conformation of the loop.

**Figure 4:**
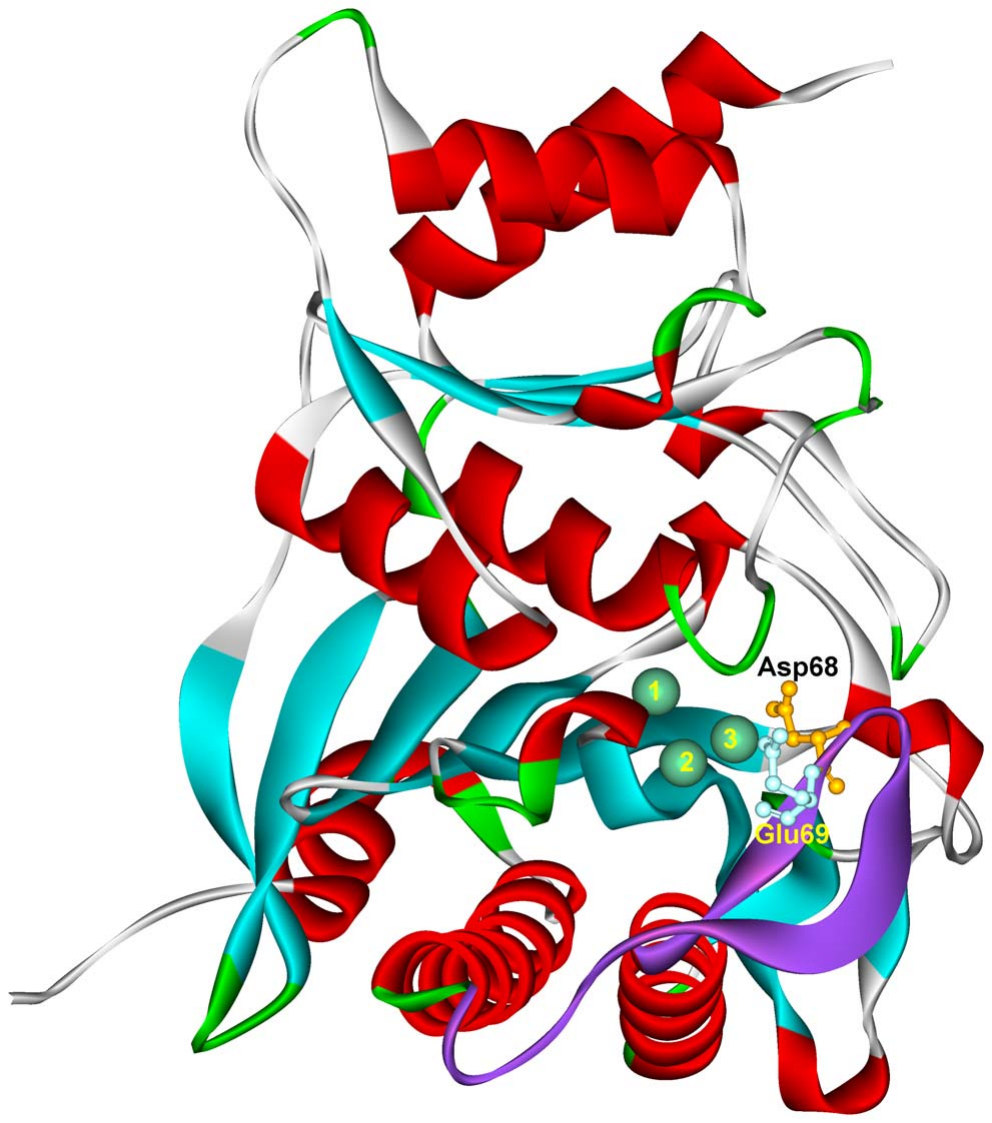
Relationship of loop 52–72 to the three divalent metal binding sites. In the engaged conformation of the loop (purple), Asp68 and Glu69 are in the close proximity to the catalytic metal binding site 3 (green sphere marked as “3”). The structure of human muscle FBPase with the loop in its engaged state was constructed on the basis of 1CNQ [23] as described by Rakus at al [11]. The image was drawn with Accelrys Discovery Studio software (Accelrys®).

**Figure 5:**
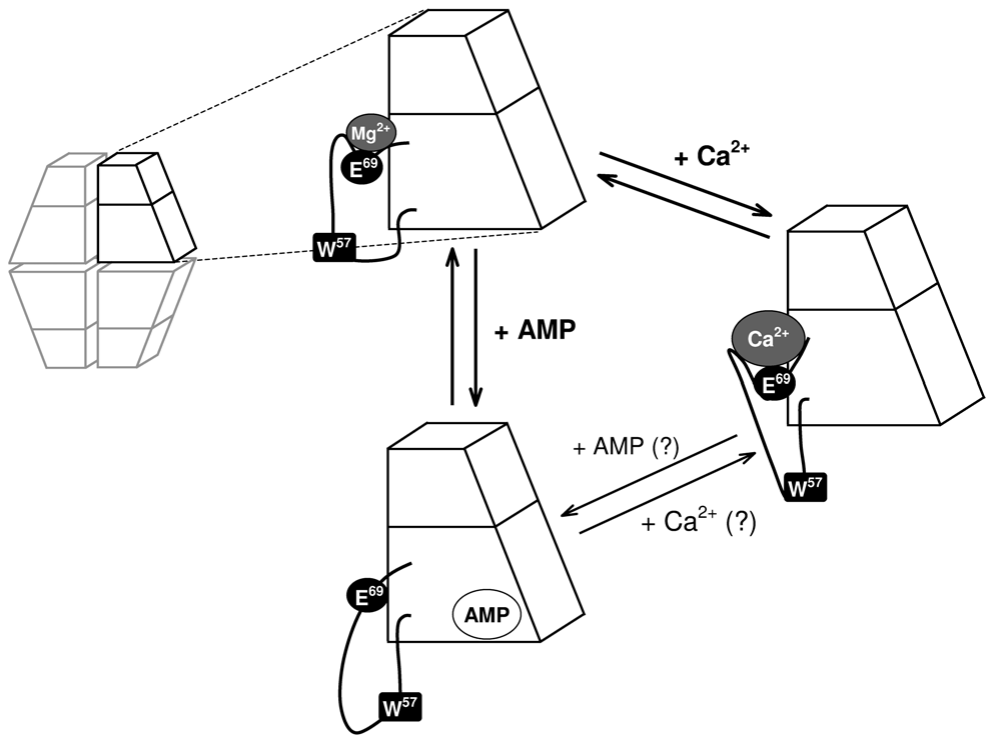
The effect of Mg^2+^, Ca^2+^ and AMP on the conformation of loop 52–72. Magnesium cations bind and/or stabilize the engaged form of loop 52–72 of FBPase, whereas association of AMP induces changes leading to the disengaged form of the loop. Ca^2+^ competes with Mg^2+^ for the same binding site and stabilizes an inactive disengaged-like conformation of loop 52–72. It is unclear whether Ca^2+^ may bind to the enzyme which is saturated with AMP and *vice versa*.

Crystallographic studies revealed that the association of divalent cations with liver FBPase occurs only if loop 52–72 is in its engaged conformation, and the residues neighboring glutamic acid 69 interact with the active center of the enzyme (Fig. 4) [22]. Thus, assuming that residue 69 is required for a strong association of both Ca^2+^ and Mg^2+^ with muscle FBPase [34], it might be expected that, like in the presence of Mg^2+^, in the presence of Ca^2+^ the loop adopts, its engaged or engaged-like conformation (Fig. 5).

However, our of fluorescence studies suggest that Ca^2+^ depopulates the loop 52–72 structure toward its disengaged conformation rather than mimicking the effect of Mg^2+^ or Zn^2+^. Considering that the fluorescent properties of Ca^2+^- and AMP-saturated FBPase are similar, and that a strong association of both Ca^2+^ and Mg^2+^ with the muscle enzyme requires the same residue (i.e. glutamic acid 69), the Ca^2+^-stabilized inactive conformation of loop 52–72 should differ from the canonical disengaged and engaged forms. Calcium ionic radius is nearly 40% larger than that of magnesium (114 Å versus 84 Å, respectively), and thus it may prevent proper association of the loop with the active site. It could be presumed that, in the presence of Ca^2+^, residues 69–72 adopt an engaged-like conformation with Ca^2+^ partially occupying the catalytic metal binding site but not supporting catalysis, while residues 52–68 adopt a disengaged-like conformation (Fig. 5). Such a mode of interaction between the cation and the enzyme implies that the T-state-like tetramer arrangement is not required for the inhibition of FBPase by Ca^2+^.

Interaction of muscle aldolase with muscle FBPase desensitizes the latter enzyme to the inhibition by AMP and, partially, by Ca^2+^ [11], [25], [35]. This interaction is stabilized by Mg^2+^ whereas Ca^2+^ disrupts it. Since Ca^2+^ prevents the formation of the active, canonical engaged conformation of loop 52–72 and Mg^2+^ stabilizes it, it is likely that aldolase binds to the active form of muscle FBPase.

Here, we demonstrate that in the presence of 10 µM Ca^2+^, which completely inhibits the wild-type muscle FBPase and disrupts its interactions with sarcomeric structures and aldolase, the Tyr^57^Trp mutant is fully active and associated with the Z-line. Only at a Ca^2+^ concentration capable of inhibiting the Tyr^57^Trp mutant (200 µM) its binding to the Z-line-based complex can be destabilized (Fig. 3; Fig. S1). These results appear to corroborate our hypothesis that aldolase associates with the active form of FBPase, i.e. the form with loop 52–72 in the engaged conformation.

Previously we showed that, unlike Ca^2+^, AMP was not able to overcome the activation of muscle FBPase by aldolase [11]. According to fluorescence studies in the current work, both the inhibitors prevented the association of loop 52–72 with the active site but it appears that the mechanism of stabilization of the inactive conformation was different. Most likely, Ca^2+^ prevents proper association of the loop with the active site by replacing the activatory cation, whereas the inhibition of FBPase by AMP results from long-distance changes within the monomer and tetramer that stabilize loop 52–72 in its disengaged conformation.

The studies of Fromm’s group revealed that AMP ligation to the R-state of FBPase induces a transition of the enzyme to the T-state, and the T-state arrangement of subunits favors the disengaged conformation of the loop [36]. Since AMP does not affect the interaction of FBPase with aldolase, it could be hypothesized that aldolase associating with the R-state blocks the T-state the transition and therefore, eliminates the ability of loop 52–72 to adopt the disengaged conformation.

Our findings provide several lines of evidence that Ca^2+^ inhibits muscle FBPase competitively to the activatory action of Mg^2+^, by stabilizing the disengaged-like conformation of loop 52–72. The results of in situ studies demonstrate that aldolase associates with the active form of muscle FBPase, i.e. with loop 52–72 in the engaged conformation, and that Ca^2+^-induced destabilization of the aldolase-FBPase complex results from depopulation of the engaged towards the disengaged-like form of the loop.

To summarize, we propose a molecular mechanism of muscle FBPase inhibition and FBPase-aldolase complex regulation by calcium ions – the processes that together comprise a key and universal cellular mechanism of regulation of the glyconeogenic metabolon activity in striated muscles.

## Supporting Information

Figure S1 **Ca^2+^-induced dissociation of FBPase from sarcomeric structures is not a result of destabilization of aldolase binding to these structures.** In the presence of 200 µM Ca^2+^, binding of the FITC-labeled Tyr^57^Trp FBPase mutant to sarcomeric structures is disturbed (A) whereas aldolase still localizes on the Z-line (B). Bar = 5 µm.
(DOC)
