# Supplementary material for: The Mechanism of Calcium-Induced Inhibition of Muscle Fructose 1,6-bisphosphatase and Destabilization of Glyconeogenic Complex
Source: PLoS One. 2013 Oct 11;8(10):e76669. doi: 10.1371/journal.pone.0076669 (PMC3795747; doi:10.1371/journal.pone.0076669)

# Supporting Information

To demonstrate that the calcium-induced dissociation of FBPase from sarcomeric structures is not a result of destabilization of aldolase binding to these structures, muscle fibers incubated in the presence of 200 µM Ca^2+^ with FITC-labeled Tyr^57^Trp mutant were fixed and stained with anti-aldolase primary antibodies and secondary antibodies conjugated with TRITC.

As it is shown in Figure S1, during the protein-exchange experiment aldolase remained bound to the Z-line.

###### Fig. S1.

**Ca^2+^-induced dissociation of FBPase from sarcomeric structures is not a result of destabilization of aldolase binding to these structures.**

In the presence of 200 µM Ca^2+^, binding of the FITC-labeled Tyr^57^Trp FBPase mutant to sarcomeric structures is disturbed (A) whereas aldolase still localizes on the Z-line (B). Bar = 5 μm.


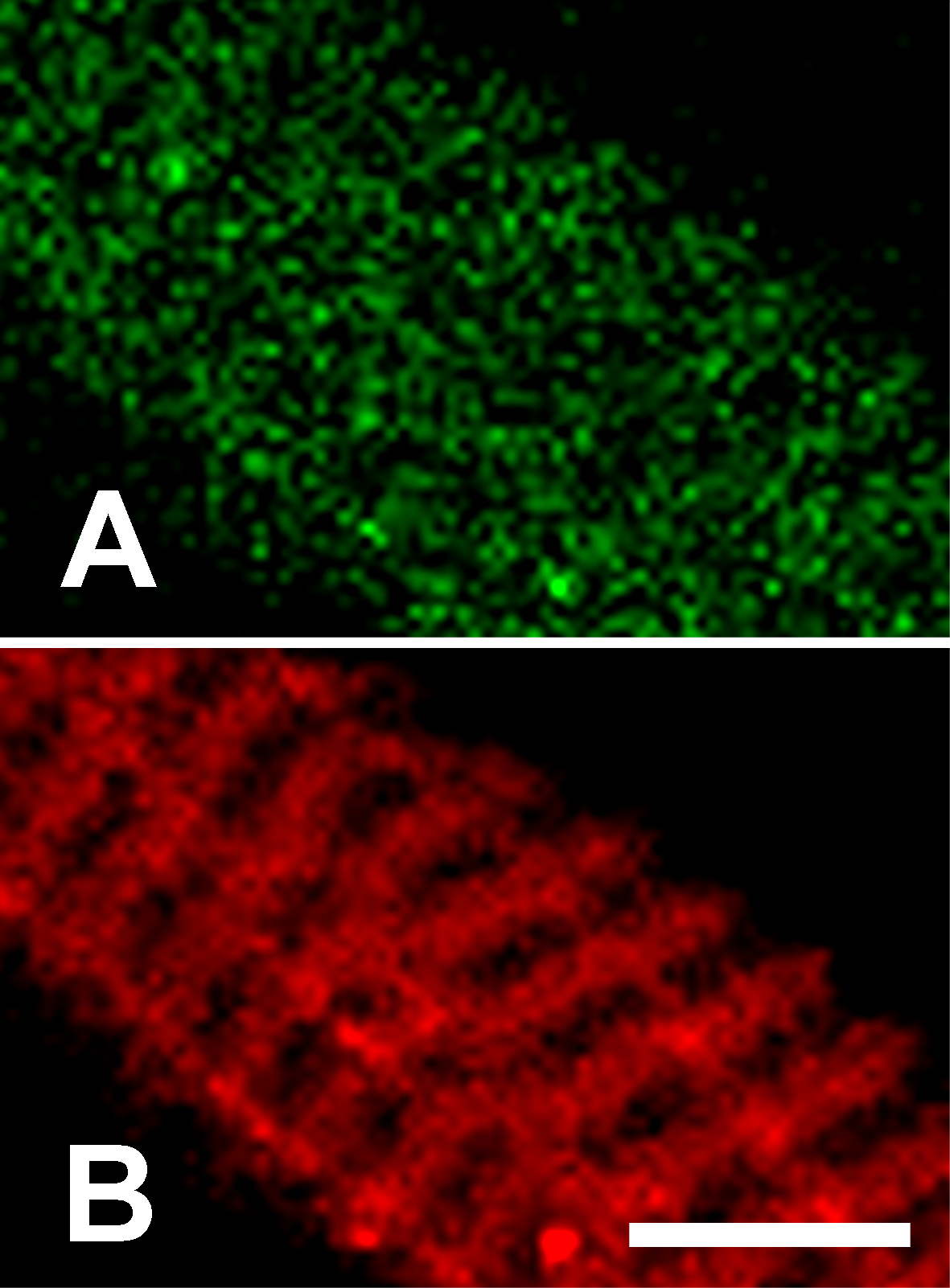

Supplement: Figure S1 — Ca2+-induced dissociation of FBPase from sarcomeric structures is not a result of destabilization of aldolase binding to these structures. In the presence of 200 µM Ca2+, binding of the FITC-labeled Tyr57Trp FBPase mutant to sarcomeric structures is disturbed (A) whereas aldolase still localizes on the Z-line (B). Bar = 5 µm. (DOC) [file pone.0076669.s001.doc]
